# Perspectives of Aboriginal People Affected by Cancer on the Need for an Aboriginal Navigator in Cancer Treatment and Support: A Qualitative Study

**DOI:** 10.3390/healthcare11010114

**Published:** 2022-12-30

**Authors:** Rosalie D. Thackrah, Lenelle P. Papertalk, Karen Taylor, Emma V. Taylor, Heath Greville, Leanne G. Pilkington, Sandra C. Thompson

**Affiliations:** 1 Western Australian Centre for Rural Health, University of Western Australia, Crawley, WA 6009, Australia; 2 Cancer Network WA, North Metropolitan Health Service, Verdun Street, Nedlands, WA 6009, Australia; 3 Aboriginal Health Cancer Lead–WA Cancer Network, Aboriginal Health Strategy, South Metropolitan Health Service, Murdoch, WA 6150, Australia

**Keywords:** Aboriginal Patient Navigator, rural and remote health, cultural safety, Indigenous health, cultural diversity, cancer care

## Abstract

Aboriginal and Torres Strait Islander Australians suffer higher rates of cancer and poorer outcomes than the wider population. These disparities are exacerbated by rurality and remoteness due to reduced access and limited engagement with health services. This study explored the cancer journeys of Aboriginal patients and carers, and their views on the establishment of an Aboriginal Patient Navigator role within the Western Australian healthcare system to support cancer patients and their families. Sixteen Aboriginal participants were interviewed either face to face, by telephone, or via video conferencing platforms. The interviews were then recorded, transcribed, and thematically analyzed using standard qualitative techniques. Close consultation within the research team enhanced the rigour and robustness of the study findings. Patients and carers identified many gaps in cancer service delivery that made their experiences stressful and unnecessarily complex. Challenges included a lack of stable accommodation, financial burdens, constant travel, being “off-Country”, and miscommunication with health professionals. Key sources of support and strength were the centrality of family and ongoing cultural connectedness. All participants were supportive of an Aboriginal Patient Navigator role that could address shortfalls in cancer service delivery, especially for patients from rural and remote communities. A culturally safe model of support has the potential to increase access, reduce anxiety and improve health outcomes.

## 1. Introduction

The incidence of cancer among Aboriginal and Torres Strait Islander Australians remains stubbornly high, with the age-standardized incidence rate for all cancers being 14% above that for the wider population [1]. Cancer survivorship rates are also poor. In 2015–2019, the mortality rate in Australia’s Indigenous population was 45% higher compared to that in the non-Indigenous population [2]. Furthermore, while falls in cancer mortality of 10% were recorded in the wider population during this period, a 12% increase occurred in the Indigenous population [3]. Despite evidence of the under-reporting of population and health data among Indigenous peoples worldwide, the disparities observed in Australia are consistent with those reported globally and have led to the prioritization of cancer research in these populations [4]. Recent research has focused on the impact of culture on cancer care, including patients’ experiences of care and the role of Indigenous researchers and communities in improving their cancer outcomes [4].

In common with similar colonial settler societies, Indigenous populations in Australia were displaced from traditional lands and discouraged from maintaining cultural beliefs and practices. In addition, many children were removed from their families and raised in institutions, a practice that did not end until the 1970s [5]. Intergenerational trauma resulting from past injustices, racism and cultural dislocation has had long-term health consequences and is largely responsible for unacceptable health disparities between Australia’s Indigenous populations and the wider population [5]. These disparities are exacerbated by rurality and remoteness; this issue is recognized in the Western Australian (WA) Cancer Plan (2020–2025) and the WA Country Health Service Cancer Strategy (2017–2022), which prioritize Indigenous populations and others living in remote locations [6,7]. Variable access to timely, quality health services is a known contributor to health disparities in rural communities where workforce issues, cultural factors and the tyranny of distance are impediments to care [6]. Efforts to improve cancer outcomes for these population groups have focused on building the Aboriginal healthcare workforce and addressing access and cultural awareness gaps.

While cancer services in regional areas of WA have increased in range and capacity over the last decade, the specialized nature of many diagnostic and treatment services frequently requires relocation to Perth, the capital of WA, which is in the southwest of the state. For many Aboriginal people, this is a daunting prospect. Reluctance to travel has been associated with a range of factors, including being “off-Country” [8]. The concept of “Country” is closely associated with traditional lands, identity, family, and community; being “off-Country” due to relocation can disrupt cultural ties, which are vital to well-being. Other factors that can affect decision-making around relocation include a fear of the city, limited logistical and financial support, the navigation of highly medicalized settings and inadequate cultural security in health service delivery [8,9]. The cancer treatment journey is a complex one for all patients; for many Aboriginal patients the challenges are multi-faceted due to poorer health status, co-morbidities, later diagnosis, socio-economic disadvantage, inequitable access to services, and, significantly for some, an underlying mistrust of the healthcare system [10].

In the United States and Canada, navigator roles exist to assist many underserved minorities who suffer stark inequities in health service delivery. The navigation concept was devised in the 1990s in Harlem, by Dr Harold Freeman, as a “person-centred approach to empower individuals and families to establish a connection with appropriate services” [11] (p. 200). Originally intended to improve marginalized women’s access to healthcare services, patient navigator programs evolved to support diverse population groups, and largely focus on cancer care. There are promising signs that patient navigators can reduce cancer health disparities by providing patients with support and guidance and improving the timeliness of access to medical services [12]. Programs have expanded over time, with navigator roles incorporating a greater range of functions, from prevention and early detection to advocacy and treatment support, as well as survivorship and quality of life [13]. Navigation roles vary, with lay navigators employed for their personal expertise and cultural knowledge, while professional navigators often have more familiarity with the healthcare system and the health issues experienced in their communities. Consequently, it has been suggested that navigator roles should not be too narrowly defined; rather, they should be integrated into a cohesive model of care, from community engagement to survivorship or end-of-life care [14]. An important strength of these programs is that patient navigators usually come from the communities that they serve; being embedded in the community likely increases the identification of local barriers to care and improves patients’ trust and adherence to recommended treatments.

The employment of Indigenous Patient Navigators who work specifically with Indigenous individuals and families has been found to be effective in building bridges between these populations and the Western healthcare system and improving patient satisfaction, although less is known about how they improve health outcomes [11]. Whop and colleagues [15] reviewed research on successful Indigenous Patient Navigator programs in the United States and found that the navigator role provided a culturally competent service to reduce cancer mortality, by means of increased screening and assisting with follow-up care coordination. Success was linked to the development of personal relationships with the participants and the emotional support that was provided. Practical support, which included cancer education and resources, discussion of treatment options, assistance with transport and daycare, financial support, and facilitating communication with healthcare professionals, was also a cornerstone of the programs [15]. Cancer Care Ontario’s network of 10 Indigenous navigators, reviewed by Sheppard and colleagues, was found to provide similar services. The authors note the importance of meeting cultural and spiritual needs, facilitating traditional healing, and providing opportunities for cultural education [16].

Rankin and colleagues’ scoping review of the role of Indigenous Patient Navigators commented on the dearth of literature from Australia and New Zealand surrounding this role, despite their colonial settler histories [11]. However, several small-scale studies in Australia have identified issues that a patient navigator could potentially address. Segelov and Garvey [4] found the psychosocial aspects of care to be critical to improving cancer care outcomes, while Bernardes and colleagues [17] observed that unmet social support needs or dissatisfaction with the support received by Indigenous patients were predictors of distress and worry about cancer. They noted that the presence of family, good social support and culturally sensitive health professionals buffered distress and alleviated anxiety, and they saw a role for patient navigators in these settings. A small experimental study, conducted by Valery [18], found that patients appreciated having access to a hospital-based Indigenous Patient Navigator; the navigator provided company during treatment, someone to talk to, and someone who showed compassion. The range of difficulties confronting Australian Indigenous patients on their cancer journeys has been widely reported upon, with miscommunication and a lack of coordination of care being regularly cited [19,20]. Regional and remote patients suffer the added burden of regular travel, being ”off-Country” and being separated from family due to the limited services available in the regions [7,8]. Cultural safety and strong therapeutic relationships have been central to reports of positive experiences of care [19,21] and it has been suggested that an Indigenous Patient Navigator would be well-placed to address these needs.

This paper reports on a qualitative study that explored the experiences of Aboriginal cancer patients and/or family members and carers regarding their cancer journey, with the aim of informing the development of an Aboriginal Patient Navigator (APN) model to operate in metropolitan Perth, Western Australia. The intent was to describe cancer experiences and identify current service needs, barriers to access, and gaps in service delivery. Patients’ views on the design and value of a proposed navigator role were also sought. If implemented, an APN role would support new Aboriginal Cancer Nurse Coordinator positions in metropolitan Perth and the Mid West, Goldfields, Pilbara, and Kimberley regions of Western Australia.

## 2. Methods

### 2.1. Design and Recruitment

The study commenced in February 2021 and involved consultations with Aboriginal people and Aboriginal health services. We also utilized the Cancer Council of Western Australia’s Aboriginal Advisory Group to discuss the aims and gain insight and advice. The 12-month timeframe was extended by 6 months, due to the impact of COVID-19 in the community and vaccination rollouts; both adversely affected the workload of the Aboriginal health services that were the proposed sites of recruitment. The aim from the outset was to build research capacity in the Aboriginal staff of Aboriginal services; however, recruitment proved difficult to achieve via Aboriginal primary healthcare and support services, despite some training to build understanding and capacity to undertake the research. Therefore, the involvement of Aboriginal research assistants within the Western Australian Centre for Rural Health was instrumental to the research process. A snowball method was employed and the recruitment of participants was facilitated by their extensive family and social networks. Approaches were also made to Aboriginal Medical Services and other agencies supporting Aboriginal patients, including accommodation services in Perth. Participants were selected for interview if they were Aboriginal and a resident in or had travelled to Perth for diagnosis or treatment (new or recurrent) in the previous three years, or if they were caring for a person who met these criteria. The aim was to ensure that participants came from a range of geographical areas of WA. Data collection ceased when saturation was achieved.

### 2.2. Data Collection, Analysis, and Rigour

Interviews were conducted between August 2021 and August 2022, and employed a range of techniques including face-to-face, telephone or videoconference platforms, depending on the participant’s preference. A semi-structured interview schedule designed by the research team was used by the interviewers; this included a targeted question about an Aboriginal navigator role. Further areas that were explored included patients’ and carers’ experiences surrounding diagnosis and treatment, assistance with accessing care, sources of support, and the challenges encountered along the cancer journey. Interviews ranged in length from 30 to 90 min, with longer interviews being conducted over several days. Interviews were conducted by most members of the research team, which was located in both Geraldton (the Mid West) and Perth. The team included two Aboriginal researchers. Participants were given information and consent forms, with consent acknowledged either by signature or when recorded verbally at the commencement of the interview. Ethics approval for the study was granted by the Western Australian Aboriginal Health Ethics Committee (11/06/2021/1053).

All interviews were recorded with permission and then transcribed either manually or using the Otter AI software. The latter process required considerable editing to make corrections. Audio files were shared among the research team, with their security safeguarded in password-protected computers. Standard qualitative techniques were employed to analyze the data: multiple readings of the transcripts to identify codes and categories of information, the development of emerging themes and extensive consultation within the research team [22,23]. A key characteristic of the thematic analysis was the regular discussions held following independent transcript readings; this ensured that the Aboriginal researchers’ interpretation of the findings and understanding of context fed directly into the development of themes and framing of the analysis. Collaboration within the research team enhanced the trustworthiness of the findings and the rigour of the analysis and accorded respect to the Aboriginal researchers’ expert knowledge.

Finally, it is important to note that throughout the data collection phase, researchers were cognizant of the need to mitigate adverse effects on the participants. Despite giving their approval to be interviewed, many were concurrently undergoing cancer treatment and suffering side effects, in addition to other physical illnesses and socio-emotional challenges in their lives. To reduce the risk of harm, participants were reminded of their right to leave the study at any time or to curtail an interview for any reason. Where participants were not known to the interviewer, efforts were made in advance to establish trust. These strategies comply with the National Health and Medical Research Council’s guidelines for Aboriginal health research [24].

## 3. Results

### 3.1. Participant Characteristics

Of the 16 study participants, 11 were cancer patients, two of whom were also caring for a family member with cancer, while five were carers of family members or friends who had cancer. All carers were female, and four of the 11 patients were male. Participants’ ages ranged from their 30s, through to their 70s, and they came from a wide range of locations throughout the state: Perth/Mandurah (5), the Mid West (Geraldton, Mullewa) (7) and the Gascoyne (Carnarvon, Shark Bay), Great Southern (Albany), and Pilbara (Wickham) (4) areas. Several participants relocated to Perth during their illness, to be closer to family and health services. Four participants had a background in working in health services.

The range of cancers included breast, cervical, oesophageal, leukemia, brain, bowel, bladder, and squamous cell carcinoma. Significantly, most patients had one or multiple co-morbidities. These included diabetes, cognitive impairment, Parkinson’s disease, and depression and/or anxiety. Cancer treatments varied according to diagnosis and prognosis, with surgery, chemotherapy, radiotherapy, and immunotherapy being the most frequent types. Patients were required to undertake their treatments in Perth, with public hospitals used by the majority; three patients attended both public and private hospitals to access treatment. Several patients utilized regional palliative care services upon returning to their communities. It is noteworthy that, in addition to mainstream medicine, many patients also embraced traditional bush medicines, lifestyle changes, and complementary medicines and practices.

### 3.2. Experiences with Cancer and Reflections on the Aboriginal Patient Navigator Role

The findings present participants’ responses to the questions posed in the interview schedule; these questions were designed to explore participants’ experiences with cancer and their views on a navigator role. Analysis of these findings produced two broad and overlapping themes: diagnosis and the coordination of care, and the centrality of family and cultural connectedness. Clarity of communication, which was an integral component of both themes, is presented in context.

#### 3.2.1. Diagnosis and the Coordination of Care

The response to a cancer diagnosis was universally met with shock and fear. Occasionally, participants spoke of missed opportunities for earlier diagnosis, such as ignoring warning signs or screening appointments, but, more often, reference was made to difficulties accessing health services in rural and remote areas. Long GP waiting times, high turnovers of staff, and the costs associated with travel for follow-up were all factors that influenced the provision of timely care. A cancer diagnosis was often associated with a death sentence and, for those in rural areas, with a fear of dying “off-Country”:

*When they told me what they detected, I started freaking out and before long I was planning my funeral.*

(Patient)

*It was an incredible shock; the doctor came out and said, ‘Oh, you’ve got cancer’… and it was in front of about a dozen people. My daughter said, ‘What happened to confidentiality?’*

(Patient)

Communication and an explanation of test results were sometimes handled poorly, from the extreme example above, of a diagnosis delivered in a waiting room, to rushed appointments with little time to fully absorb the information relayed. Fear also impacted the processing of information. While the use of medical language in consultations was frequently mentioned as a barrier to understanding, some participants provided examples of skilled communicators who visually represented the disease process and the steps that lay ahead:

*That the doctor drew a picture made a huge difference… that first initial breakdown, talking to me, showing it to me… because my brain was still trying to process that I’d just gotten this bad news… you only hear a small amount of what’s being said.*

(Patient)

Most patients were accompanied by a family member or friend when they received their diagnosis, although, occasionally, a deliberate choice was made to attend alone and protect loved ones from bad news. During the treatment phase, however, support networks were important sources of emotional and practical support for patients.

While participants’ experiences with cancer services and the coordination of care varied according to geographical location, knowledge of the health care system, financial circumstances, and family support, rural patients identified common challenges that were due to relocation. These included access to stable accommodation, family and transport arrangements, additional costs, and, for some, a sense of uncertainty and confusion arising from the fast-paced city environment. Rural patients can access the government-funded Patient Assisted Travel Scheme (PATS) to offset transport costs, but, regardless, regular travel over vast distances incurs costs over and above the assistance provided. Issues surrounding accommodation were raised more often, however, especially among those who required extended stays in Perth. While several providers offer short-stay accommodation close to the treating hospitals, living arrangements for others were often fraught:

*Living arrangements in Perth were difficult; City Stay apartments, then Coolbellup, the Allawah, then Maylands, then Crawford Lodge … and being off-Country was hard.*

(Carer)

*Accommodation was all over the place … accommodation for independence is the main thing, preferably close to the hospital.*

(Patient)

One carer drew attention to the challenges facing Aboriginal patients from remote areas. These included being “off-Country”, limited understanding of the diagnosis and treatment plan, treatment in Perth being associated with dying, and a history of mistrust of service providers. The link between being “off-Country” and dying has particular significance, as many Aboriginal people wish to die “on Country”. One carer explained how she advocated for patients; in addition to attending consultations, she later relayed in plain language the information imparted in the consulting room:

*I’d speak to the doctor … and later describe the cancer like a tree, breaking down information so people can understand, trees grow roots, translate it to mother nature… this is the key issue, making it more easily understood … turning it into something from the land is what I try to do.*

(Carer)

While participants often spoke highly of the care received, a breakdown in communication with healthcare professionals was a common occurrence. Participants noted the limited availability of Aboriginal Health Liaison Officers (AHLO), who were sometimes able to do little more than introduce themselves and were rarely involved in the patient’s care or providing specific input and advice. In these circumstances, family members and advocates with knowledge of the healthcare system were huge assets for patients. Interestingly, some participants themselves observed glaring gaps in service delivery for others while awaiting their own treatment, and occasionally intervened:

*There was an old Aboriginal lady on the chair next to me, and they were having a hard time trying to find a vein for her. And they were trying to convince her to have a port and she didn’t want the port. She didn’t understand. I said to her when the nurses had left. Look, excuse me, but I’ve got a port and it’s the best thing I’ve ever done. And she said, does it hurt, and I said no, you’re asleep when they put it in.… when she was leaving, she said, can I have a look at your port? It was all about explaining… she was frightened.*

(Patient)

Other challenges identified by participants included the number of hospitals that the patients were required to attend and instances of a breakdown in communication between the various settings, the late rescheduling of appointments, especially for country patients, dealing with missed appointments, and poor discharge planning. For some participants, both patients and carers, the healthcare system was just too complex and difficult to navigate and became an added source of stress and anxiety.

#### 3.2.2. Centrality of Family and Cultural Connectedness

In most cases, family members were referred to as the main source of strength and emotional support. Frequently, they were relied upon to access information, assist with securing accommodation, handle medications and food, arrange travel, and update extended family members on a patient’s progress. Furthermore, family members were frequently carers; this came at a cost, as many carers themselves had existing health problems. Others were required to take extended leave from employment to support a family member undergoing treatment in Perth. While family dynamics had to be negotiated, patients referred to regular family visits as being essential to their well-being. Carers spoke of their advocacy role and the need to be there to intervene on behalf of their family member. A striking example of the important role that family played beyond emotional and logistical support is provided below, where the insistent demands of an elderly Aboriginal woman’s daughter ensured that she received emergency care before there was a serious adverse outcome.

*It was scary… they tried unsuccessfully three times to do this procedure… the third time was down at xx Hospital… but what happened, that didn’t work, they collapsed Mum’s lung. And she almost died.*

*So, at the time, there was probably about four other code blues happening. I was in the waiting room, I took her in, and they brought her back. And I knew immediately… my mum never complains, she’s as tough as … she was going like this and rocking in the bed. And I knew that something serious was wrong and so I called for a nurse … she came back and she’s sorry, there’s four code blues happening, which are urgent situations. And I said, well, you’re gonna have another one here on your hands up, you know, come and see to her, it was chaos. Absolute chaos there.*

*Anyway, she went away, and she said that the doctors would be with me in a few minutes. This point, she started going downhill really badly. I was watching the monitors; everything was going pear-shaped. So, I ran to find that nurse and she’d got distracted with another code blue. And I actually had a big growl at her… within five minutes, I had the head specialist, and head surgeon and other specialists and said, yes, they said, her lung has collapsed, we got to get her down to emergency surgery, which they did. And thankfully, she pulled through that.*

(Patient carer)

In addition to family members, participants identified other sources of support and information including the internet, cancer nurses, dietitians, members of the Cancer Council of WA, and staff at the accommodation facilities.

The importance of culturally safe care and remaining connected to the community were issues frequently raised by participants. Patients and carers provided numerous examples of healthcare delivery that was considered culturally unsafe. Of note was intimate care being provided to Aboriginal women by male health workers, which was a source of great embarrassment. On one occasion, when a male nurse was sent to bathe an elderly Aboriginal woman, a family member intervened due to her distress. The presence of male-only radiographers during breast scans was also seen as culturally inappropriate. Significantly, several participants suggested that a male Aboriginal Health Liaison Officer would also be unwelcome, such is the propriety surrounding Aboriginal “women’s business”. Another source of cultural distress was being “off-Country” and away from community; the impact of cultural dislocation and the yearning for Country and cultural connections often went unrecognized by healthcare workers:

*Someone who was well-informed and understood Aboriginal culture would have made a huge difference at times … I felt so despairing. Having that extra support would have made a world of difference to me… it was isolating and lonely.*

(Carer)

Conversations around cultural issues in care also revealed the widespread use of traditional bush medicines by Aboriginal cancer patients. All reported their beneficial impact but only rarely was information about usage shared with the treating doctors:

*When he went to hospital, he took a big bottle of bush medicine… and the doctors are giving him medication, not realising he was taking bush medicines, he didn’t feel comfortable about raising it… I think acknowledging Aboriginal peoples’ spirituality too could really help with mental health issues.*

(Carer)

While culturally safe care was sometimes absent and communication barriers were identified, most participants described their interactions with healthcare staff as being positive. On one occasion, racist behaviour was encountered and reprimanded; however, bad experiences were the exception. Despite this finding, it was widely recognized that Aboriginal people’s cancer journey experiences would improve if health professionals had a deeper understanding of Aboriginal family structures, the significance of Country and the impact of dislocation, the role of spirituality, and the widespread use of bush medicines. It was also believed that a better appreciation of patients’ social circumstances would enhance patient care.

#### 3.2.3. Participants’ Reflections on an Aboriginal Patient Navigator Role

All participants considered that an Aboriginal Patient Navigator had the potential to improve Aboriginal patients’ cancer journey experiences and address gaps in service delivery. Cultural knowledge was highlighted as a strength and advocacy, coordination of care, and education were important functions:

*They need an advocate to ensure patients’ voices are heard, articulating their needs… a navigator could help with your care plan and move around the hospital, help simplify language. Relationships are important … they would be a link between the family and the hospital… and then there’s cultural knowledge, knowledge of where people come from and how they’re connected.*

(Patient)

Unpacking fears around diagnosis, translating medical jargon into language that patients understand, and reminders about pre-treatment requirements were also raised as potential benefits. It was recognized that currently, AHLOs perform some of these functions, but since few participants had contact with AHLOs, an additional role that was integrated into the care team was seen to be desirable. It was noted by some that to be successful, the role needed to be clearly explained to the treatment team since experience had shown that Indigenous knowledge is not always respected: “Knowledge of communities needs to be accorded worth, respect, the same as clinical knowledge”. Other insights offered about the role include a capacity to connect virtually to absent family members, an opportunity for patients to build a relationship with one person across their cancer journey, and the initiation of conversations around discharge planning and palliative care. Lastly, it is noteworthy that most female patients indicated that they would feel uncomfortable with a male Aboriginal Patient Navigator.

## 4. Discussion

Aboriginal and Torres Strait Islander populations suffer higher rates of cancer and poorer survivorship outcomes than the wider population [1,2]. These disparities are exacerbated by rurality and remoteness and have led to the development of cancer strategies in Western Australia that prioritize these populations and others living in rural and remote locations [6,7]. There are numerous impediments to care for Aboriginal patients and their cancer journeys are often complex. In addition to the problems surrounding timely access, travel, and cultural safety in service delivery, many patients present with co-morbidities and have limited logistical and financial support [8,9,10]. In similar post-colonial settings, a navigator role has shown promise in addressing health disparities in underserved minorities by improving access to services and supporting patients via advocacy and education [11].

This study explored the cancer journeys of Aboriginal patients and carers and their views on a new Aboriginal Patient Navigator (APN) role that is proposed within the Western Australian healthcare system. Due to disruptions caused by COVID-19 and the extra caution exercised by participants, many planned interviews were cancelled due to difficult timing and illness and/or tiredness following treatment. Despite this, those who participated in the study provided rich insights into the challenges they faced during their cancer journey, what had helped them to overcome these challenges, and how an APN might support families and address gaps in service delivery. Two overlapping themes arose from these insights: diagnosis and the coordination of care, and the centrality of family and cultural connectedness; issues related to communication were evident across both themes.

### 4.1. Diagnosis and the Coordination of Care

The participants exposed many gaps in the coordination of care, including securing and getting to appointments, arranging travel for treatment upon relocation and between various health service settings, and securing stable, extended-stay accommodation in Perth. These are not new challenges and have previously been extensively described. Over a decade ago, Shahid and colleagues conducted interviews with rural Aboriginal cancer patients and revealed how transport and accommodation difficulties serve as a deterrent to accessing care and influence the patients’ engagement with the healthcare system [8]. Meiklejohn et al. [25], who reported on health professionals’ perspectives on barriers to Aboriginal cancer care, also highlighted issues surrounding the coordination of care, suggesting that those delivering services need to be cognizant of the difficulties experienced by patients. More recently, Green and colleagues identified the coordination of care and transition between services as critical in shaping Aboriginal cancer patients’ experiences of care and drew attention to the importance of Aboriginal staff and cultural safety as enablers of positive experiences [20].

In this study, the complexity of the health care system and limited continuity of care added another layer of confusion and distress, especially for some older, rural, Aboriginal cancer patients who had minimal social support. While AHLOs can address some of the needs identified, their numbers are small, and their input is required across many departments and areas of the hospital. Participants had little interaction with AHLOs, yet it has long been known that Aboriginal patients in hospital want Aboriginal people to be involved in their care and that unmet social support needs exacerbate stress and worry [9,11,14]. Issues identified around the coordination of care and frequent references to loneliness and isolation highlight the contribution that an Aboriginal Patient Navigator could make by interfacing with Aboriginal patients directly and linking them with their wider family, providing the anchor that many patients felt that they needed. They could also liaise with AHLOs and social workers.

### 4.2. Centrality of Family and Cultural Connectedness

Aboriginal people have consistently highlighted the importance of family and culture during periods of hospitalization [19,26,27,28]. Recent data gathered from yarning circle sessions facilitated by an Aboriginal Elder in rural Victoria reinforced earlier findings on the priority given to sick family members, often at the expense of one’s own health [28]. Ristevski and colleagues found that these roles and responsibilities resulted in family members providing critical practical support regarding travel, financial issues, childcare, and accommodation, in addition to much-needed emotional support. They noted that “culture and family were central to treatment and survivorship experiences of Aboriginal patients” [28] (p. 128). However, they observed that health professionals had a limited understanding of these cultural obligations; participants reported negative attitudes when large groups of family members visited hospitals and family members were frequently excluded from treatment decision-making [28].

Findings from this study reinforce the critical role played by family members across a patient’s cancer journey. Family members accessed resources for their loved ones, offered emotional and logistical support, and, when required, acted as advocates, intervening on behalf of the patient if necessary. It was recognized that these responsibilities incurred financial and emotional costs for family members. Carers often took unpaid leave to stay with a patient upon relocation, and, in some cases, their own health was already compromised. This burden was reported in Taylor and colleagues’ study of Aboriginal cancer patients and was exacerbated if travel from rural and remote areas was required [19].

The proximity of family is closely tied to cultural connectedness and, for rural and remote patients, having family close at hand reduced the stress associated with being “off-Country”. Participants’ beliefs about cancer and treatment were often imbued with cultural meanings that co-exist with mainstream medical practices. For example, the use of bush medicines and a physical connection with Country were described as positive experiences that aided healing. Limited understanding of cultural practices and protocols by health professionals was regularly observed. The use of bush medicines was rarely shared and, on occasion, patients’ cultural needs and circumstances were overlooked. Examples were also provided of the culturally inappropriate care of female Aboriginal patients.

### 4.3. Clarity in Communication

Miscommunication between Aboriginal patients and health professionals has the potential to adversely affect engagement and clinical outcomes and has been widely reported upon [9,20,29]. Impediments to communication occur for a range of reasons, including language barriers, the use of medical jargon, differences in communication styles, and a lack of rapport [9]. Barriers to effective communication need to be understood in the context of past policies and practices, experiences of racism, and a lack of trust, all of which continue to alienate Aboriginal people from the healthcare system [5,9]. In this study, miscommunication between health professionals and patients and their carers was a common occurrence across patients’ cancer journeys. In some instances, this was the outcome of an inability to process information due to fear and anxiety; on other occasions, communication breakdown occurred when medical language was not deciphered. Pictorial representations and plain English explanations of the disease process and treatment plan were identified as elements of good communication.

Communication styles vary, as do cultural approaches to how information is imparted. Body language, silences, gestures, and language proficiency can all contribute to miscommunication during a consultation [9]. An Aboriginal Patient Navigator would be well placed to recognize these barriers to effective communication, demystify medical language, and provide cultural input and interpretation. They could also liaise and enhance communication within the healthcare team, another area identified by participants as being in need of attention.

### 4.4. Reflections on the Aboriginal Patient Navigator Role and How It Differs from the Aboriginal Health Liaison Officer Role

Well-designed and community-informed models of patient navigation implemented internationally have successfully built bridges between underserved and culturally diverse populations and the providers of health services, resulting in improved access and levels of satisfaction with cancer care [11,12,13,14,15,16]. Indigenous Australians face similar barriers to care, including a long history of exclusion and racism. Gaps in service delivery have been well documented—and yet, a failure to deliver culturally safe care persists [8,9,10].

At the beginning of this research, a critical issue was how an Aboriginal navigator role would differ from AHLO positions, which are now well-established in tertiary metropolitan hospitals. Although there is no single model of how AHLOs function, given that they are employed within different health services, it became clear that there are distinct differences in how an Aboriginal navigator function might operate. AHLOs are largely hospital-bound and operate across multiple patients and departments. None of our participants described interactions with AHLOs wherein specific information about their disease was provided. Nor did they observe AHLOs being embedded in their care team, involved in treatment decision-making, or liaising with family members. In this study, participants’ reflections on a navigator role highlighted the importance of having an Aboriginal person connected to a patient as they progressed along their cancer treatment journey: an advocate and an anchor. While there was general satisfaction with the care provided by non-Indigenous health professionals, a sense of cultural distance remained. Most considered that they could build a stronger relationship with an Aboriginal person; this would facilitate improved communication and an ability to express unmet needs. In addition to providing a culturally safe and supportive environment, Aboriginal Patient Navigators could also be involved in staff education, addressing shortfalls in culturally sensitive and respectful patient care.

Participants made it clear that an Aboriginal navigator role would be a huge step forward in care provision; a navigator could support a patient’s care in hospital and connect with family and health services in their home community. To be successful, however, navigators would need to be integrated into the care team to ensure the two-way provision of information and advocacy for the patient, and some participants were adamant that both male and female positions were required, to accommodate cultural sensitivities. The role would need to be clearly defined, to complement the work of existing AHLOs. There is likely a shortage of AHLO positions; in addition, they appear to have multiple roles within the hospital, including training staff to develop their cultural understanding and advocating within the hospital for better systems to improve Aboriginal care. This leaves less time for them to support individual Aboriginal patients and provide input to team care-planning meetings. Navigators could be truly person-focused and provide care across the interfaces of the health system, including the provision of information and support to patients outside of the hospital, and when they return to their families and communities. In addition to being integrated into the healthcare team, with a clear delineation of their function, the success of an Aboriginal Patient Navigator role requires funding and a long-term commitment to building the Aboriginal healthcare workforce. A pilot program in one hospital, with a developmental evaluation, could inform the implementation of this role more widely.

### 4.5. Limitations

Due to various challenges, including a surge in COVID-19 cases, few participants came from the remotest areas of Western Australia, and we interviewed fewer participants than originally anticipated. Regardless, the geographical spread included diverse rural and regional locations, in addition to metropolitan Perth. Despite efforts to recruit both male and female participants, interviews with males were harder to secure and so males were under-represented, a common reality in health services research with Aboriginal people.

## 5. Conclusions

Aboriginal Australians suffer higher rates of cancer and poorer outcomes than the wider population. Rurality and remoteness and limited engagement with health services exacerbate these disparities. Furthermore, many Aboriginal cancer patients suffer ongoing socioeconomic disadvantage and multiple co-morbidities, making timely access to quality care more difficult. It is well established that Aboriginal people would prefer to have more Aboriginal health providers involved in their care and that when this occurs, their satisfaction with service provision increases.

Cancer navigator roles have been successfully incorporated into healthcare teams internationally and this study provides further evidence supporting their introduction into the Western Australian healthcare system. Patients and carers identified many gaps in cancer service delivery and numerous challenges, including a lack of stable accommodation upon relocation, the financial burden related to time away and accessing treatment, being “off-Country”, and miscommunication with health professionals. An Aboriginal Patient Navigator role, designed to complement the work of hospital-based Aboriginal Health Liaison Officers and integrated into the healthcare team, could support and provide advocacy for patients during hospital stays and upon returning to their communities. This culturally safe model of support has the potential to increase access to health services, reduce anxiety, and improve health outcomes. A carefully implemented program should include both female and male positions, to accommodate cultural sensitivities, and an evaluation component to assess the Aboriginal Patient Navigator’s contribution to improved cancer care and outcomes for Aboriginal patients.

## Data Availability

Data are available only on request due to privacy considerations.
